# Effects of Temperament on the Reproduction of Beef Cattle

**DOI:** 10.3390/ani11113325

**Published:** 2021-11-21

**Authors:** Alice Poggi Brandão, Reinaldo Fernandes Cooke

**Affiliations:** Department of Animal Science, Texas A&M University, College Station, TX 77843, USA; alicepbrandao@tamu.edu

**Keywords:** beef cattle, behavior, cortisol, production, reproduction, stress, temperament

## Abstract

**Simple Summary:**

In beef cattle and other livestock species, temperament can be considered as their behavioral responses to human interaction. Temperament evaluation allows classifying cattle according to the level of responsiveness, from calm to excitable. Consistently across studies, beef females classified as more excitable have signs of increased stress response and are less fertile compared to cohorts with calmer temperament. Cattle temperament can be improved via genetic selection and acclimating young animals to human interaction and handling procedures.

**Abstract:**

Temperament is often defined as the behavioral expression of animals in response to human interaction. Cattle temperament can be evaluated using an association of chute score and exit velocity, with cattle then classified as adequate or excitable temperament. To assess the impacts of temperament on various beef systems, these evaluation criteria were associated with productive and reproductive parameters of *Bos taurus* and *B. indicus*-influenced cattle. Consistently across studies, excitable cattle had greater plasma cortisol compared to animals with adequate temperament. Studies also reported that excitable beef females have poorer reproductive performance compared to calmer cohorts, including reduced annual pregnancy rates, decreased calving rate, weaning rate, and kg of calf weaned/cow exposed to breeding. Acclimating *B. indicus* × *B. taurus* or *B. taurus* heifers to human handling improved behavioral expression of temperament and hastened puberty attainment. However, similar benefits were not observed when mature cows were acclimated to human handling. Collectively, temperament of beef females measured via behavioral responses upon human handling impacts their reproductive and productive responses independent of breed type, and should be considered for optimal beef cattle production.

## 1. Introduction

Human temperament was first defined in ancient times. According to Hippocrates (460 BC to 370 BC), human temperament was classified as sanguine, choleric, melancholic, or phlegmatic in which people behave and react to the world, according to the influence of different bodily humors [1]. As the study of the human psych has evolved, so has the classification and understanding of temperament. Behaviors are one way to express temperament [2] and these expression—such as aggressiveness, nervousness, apathy, affection—are associated with various important traits of overall health and wellbeing for humans and animals. [3,4,5]

In livestock species, the evaluation of temperament relies mostly on their behavioral and phenotypical expressions [6]. Accordingly, temperament in beef cattle has been measured and traditionally associated with their behavioral responses to human handling [7,8,9,10]. Temperament has significant consequences on growth, health, and reproduction [4] and has interested researchers for many decades [11,12,13], becoming even more relevant in recent years due to increasing societal concerns regarding animal welfare [14].

## 2. Assessment of Temperament in Beef Cattle

While some livestock species are reared in controlled environments that reduce the need for robustness and defensive behavior, such as poultry, pork, and even dairy cows, beef cattle are still faced with environmental challenges more frequently, particularly in the cow-calf sector [15]. Even though some beef breeds have been intensively selected for docility, temperament is still variable and more temperamental animals are present in most cowherds [16,17]. In a global level, most commercial cattle are in tropical and subtropical areas, where heat and parasites are issues often addressed by increasing the inclusion of *Bos indicus* genetics. Although more resilient in the described conditions, *B. indicus* cattle have been shown to have a more excitable temperament compared to *B. taurus* breeds [10,18,19]. Thus, practical strategies to evaluate temperament and properly manage beef cattle with different behavioral responses are warranted to ensure optimal efficiency, sustainability and welfare of both cattle and handlers [14].

There are many forms of assessing temperament by evaluating behavioral responses and other traits in beef cattle. The decision on which technique to utilize will depend on the objectives of the assessment, facilities, and personnel, as well as the type of cattle being evaluated. Some examples of temperament assessments described in the literature are chute score, pen score, exit velocity, exit score, temperament score, and position of forehead hair whorl [4,20,21]. The pen score measures the direct interaction of animals with humans and is a good indicator of aggressiveness, however it is not recommended if the cattle evaluated are too excitable or if the handlers are not experienced, due to safety concerns. Pen scores may also not be feasible when utilizing grazing cattle or large groups of cattle [22]. The position of hair whorl on the forehead may be a useful strategy to visually assess temperamental tendencies, with no equipment required. Animals with no hair whorl or with a hair whorl positioned above the eye line were described to be more agitated compared to animals with hair whorls located in the center of the forehead, or below the eye line [20].

A temperament score that is calculated from the average between chute score and exit score [4] has also been extensively utilized in research studies. More specifically, the chute score is determined by observing the restrained animal in the chute and scoring its behavior according to the following scale: 1 = calm with no movement, 2 = restless movements, 3 = frequent movement with vocalization, 4 = constant movement, vocalization, shaking of the chute, and 5 = violent and continuous struggling. Exit velocity is assessed immediately after the evaluated animal is released from the squeeze chute by measuring rate of travel over a pre-determined distance with an infrared sensor. The exit velocity is then converted into a 1–5 scale by dividing animals into quintiles according to their exit velocity and assigning a score from 1 to 5 (exit score; 1 = cattle within the slowest quintile; 5 = cattle within the fastest quintile). This conversion aims to attenuate the potential interference of the environment, facility design, handlers, and other variables that may influence the speed of cattle leaving the chute [23], which allows comparisons across different scenarios and chute settings. Chute score and exit scores are then averaged, resulting in an individual temperament score between 1 to 5. Subsequently, animals are classified according to their temperament score as having *adequate* temperament (temperament score ≤ 3) or excitable temperament (temperament score > 3). This threshold was determined based on research from our group suggesting that moderate temperament (such as temperament scores 2 and 3) does not substantially impair production traits and may even be desirable to cope with environmental challenges [15]. Advantages of the temperament score system include a combination of an objective (exit velocity) and a subjective component (chute score), as well as potential adoption by commercial and research beef operations. However, as the other temperament assessment forms, this temperament score has limitations including the need to bring animals to the chute, the potential association to activities that interfere with baseline temperament expression (e.g., weaning, vaccination, breeding, etc.), and the need of proper velocity measurement equipment.

The concept of temperament—an innate behavioral pattern performed in response to a perceived threat—is physiologically comparable to the core definition of stress [24]. Cortisol is the main biomarker of stress in the blood, thus a relationship between circulating cortisol concentrations and cattle temperament has been proposed and demonstrated by various studies, in which more excitable cattle have consistently higher plasma or serum cortisol compared to calmer cohorts (Table 1, [4]). However, the collection of a blood sample from beef cattle involves a plethora of visual, auditory, olfactory, and tactile stimuli, which are, inherently, stressful events and elicit an acute increase in blood concentrations of cortisol [25,26]. Although some studies may refer to a *basal level* of blood cortisol in beef cattle, this is difficult to achieve as the sampling procedure per se induces a neuroendocrine response, unless, for example, extremely tamed and acclimated animals are utilized.

**Figure 1:**
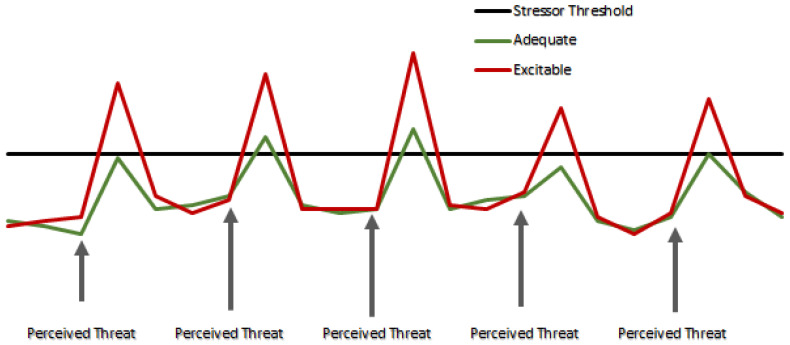
Proposed scheme of circulating concentrations of cortisol (not in scale) in excitable vs. adequate cattle in response to perceived threats (i.e., human handling for blood collection). It is speculated herein that basal levels of cortisol are similar between two temperament types, according to assessment of chronic stress via hair cortisol analysis [31]. Sampling events are perceived as threats by beef cattle, eliciting a stress neuroendocrine response. Peaks represent the blood collection moments, in which the differences between temperament types are noticed and described in literature [4,27,28,29,35,36].

As an attempt to circumvent the effects of acute stress associated with the sampling procedure, Cooke et al. [31] utilized cortisol extracted from hair as an assessment tool of chronic exposure to stress [26,37] and perhaps differences in basal levels of cortisol between *Nelore* cows classified as excitable or *adequate* regarding temperament. However, there were no differences in hair cortisol concentrations between the cows in the two temperament type groups. An explanation for such a result was beyond the hypothesis and scope of the study and further research is still warranted to elucidate these findings. One can speculate that, as their assigned name defines them, the excitable cows do not present uniformly increased concentrations of basal cortisol, but rather, respond more intensively to certain perceived threats. As proposed in the schematic Figure 1, it can be speculated that beef cows classified as having excitable temperament are not ‘*more stressed*’ animals compared to calmer cohorts, being instead ‘*more easily to become stressed*’ animals, with similar basal cortisol concentrations, if no threats are present.

## 3. Temperament, Stress, and Reproductive Efficiency in Beef Cattle

Regardless of the manner or pattern in which the stress response differs between excitable and adequate tempered cattle, the literature strongly supports that excitable cattle have higher cortisol concentrations compared to cohorts with more adequate temperament during handling procedures (Table 1). If this difference persists when animals are not being sampled is a topic that warrants further investigation. Cortisol concentrations in the blood, as the main marker of the neuroendocrine response to stress, has a negative relationship with reproduction in cattle [27] and other mammal species [38]. Aspects of personality and behavioral traits have been shown to affect fertility even in humans [39,40]. Elevated concentrations of circulating cortisol have been associated with reduced gonadotropin activity and ovarian steroidogenesis in females [41,42]. Chronic and acute stress models have been associated with impaired reproduction in beef cattle by decreasing pregnancy rates and increasing pregnancy loss [43,44].

Catecholamines have also been associated with acute stress and the so called “*fight-or-flight*” response [45]. These neurotransmitters are markers of the sympathetic division of the autonomic nervous system, which coordinates the behavioral and physiological expressions of stress in association with the hypothalamus-pituitary-adrenal axis, and is responsible for the release of cortisol and other glucocorticoids in the bloodstream [21]. Concentrations of catecholamines, particularly epinephrine, in the circulation tend to display a behavior comparable to cortisol regarding time and magnitude of its increase in the circulating concentrations. However, epinephrine is present in the blood in much lower absolute concentrations [46,47,48] which may hinder its laboratorial handling and analysis. Hence, cortisol has been more utilized as a biomarker of stress than catecholamines due to practical rather than biological reasons, [49,50,51].

Stress, cortisol, and temperament are so conceptually close that makes it difficult to separate their individual effects on cattle reproduction. However, our and another research group made significant efforts to elucidate the impacts of temperament in the fertility of beef cows [4]. Cooke et al. [34] identified a linear relationship between temperament scores assessed in the beginning of the breeding season and the probability of pregnancy in Braford and Brahman × British beef cows exposed to natural and artificial breeding. Females with more adequate temperament were more likely to become pregnant compared to more excitable cohorts. Interestingly, pregnancy rates were also negatively affected by excitable temperament in cows exposed to natural breeding only, suggesting that temperament also impacts reproductive function by other pathways besides stress from human interaction.

Data from *B. indicus* beef cows [52] corroborated with what had been reported for crossbred animals. The probability of pregnancy was negatively associated with temperament score and pregnancy rates tended to be negatively associated to temperament. Similar results were reported when *B. taurus* cows were utilized [30], in which cows with adequate temperament had higher pregnancy rates compared to more temperamental cohorts. Additionally, improved overall productivity was noted in cows with adequate temperament in both bovine subspecies when compared to their more excitable cohorts (Table 2, [30,31]). Overall, calmer cows had greater pregnancy, calving, and weaning rates, and were more efficient in terms of kg of calf weaned per cow exposed for both *B. indicus* and *B. taurus* females. In *B. indicus* females, pregnancy loss was also reduced and calf weaning weight was increased in those with adequate temperament. Perhaps these differences were due to variations in reproductive and general management between studies, such as different hormonal protocols for estrus synchronization, a different number of AI events, and different criteria for cows being inseminated versus exposed to natural service.

Besides fertility variables such as rates of pregnancy and calving, and parameters of maternal ability such as offspring growth and survival, puberty attainment of replacement heifers is also extremely impactful on the productivity of cow-calf operations [53,54]. As most biological processes, the initiation, development, and proper resumption of puberty, resulting in a sexually mature bovine female is complex and multifactorial [55]. The effects of nutritional and hormonal management on puberty attainment by beef heifers are well described [53]. However, there is evidence of temperament also playing an important role on the development of fertile replacement heifers [13]. Interestingly, initial studies investigating temperament and puberty in beef heifers focused on acclimation as an alternative to overcome negative effects of stress on puberty attainment [33], with research on the actual effects of temperament on puberty being developed only years later [32]. In contrast, the effects of stress as neuroendocrine response on puberty attainment, although not fully understood, are well described [13,24]. Many aspects deemed detrimental to puberty attainment have been associated with high levels of circulating cortisol and thus, a neuroendocrine stress response. As previously mentioned, the concepts of stress and temperament are often interweaved in their definitions, assessments, and expression by the animals, perhaps that is the reason why more specific research was not developed until recently.

Attending this research need, our group investigated the effects of temperament on puberty attainment in Nelore heifers [32]. Temperament evaluation followed similar criteria as described herein and in previous literature [4]. Based on temperament assessment, heifers were classified as adequate or excitable and evaluated for puberty attainment via verification of ovarian activity through transrectal ultrasound performed in 10-day intervals [56]. Puberty attainment was delayed in heifers with excitable temperament compared to their adequate cohorts, consequently, the percentage of excitable heifers that were pubertal by the end of the study was less compared to adequate cohorts (24.9% vs. 42.9%, respectively). Pubertal heifers with adequate temperament were also heavier at puberty, but not older, compared to their excitable cohorts due to greater average daily gain during the experiment. Increased body weight gain is known to hasten puberty in beef heifers [57] and temperament has been shown to affect average daily gain in growing beef cattle, with excitable animals consistently performing poorer compared to calmer cohorts [58,59,60]. Thus, it is difficult to determine a clear cause-effect relationship between those variables—body weight gain, temperament, stress, and puberty attainment—but a relationship among these parameters is evident, in which animals with more excitable temperament are, overall, counterproductive for the beef producing chain.

## 4. Improving Temperament to Optimize Reproductive Outcomes in Beef Operations

As it has been demonstrated herein, excitable temperament has a negative impact on beef production since it negatively impacts different aspects of reproduction, reducing profitability outputs such as calves born and weaned, weight of terminal animals, and increased time for replacement heifers to reach puberty [4]. Besides, excitable animals pose a threat to the safety of handlers and may increase the cost of maintenance in the operation by damaging elements such as fences, feed bunks, and water troughs. Alternatives to improve temperament or more efficiently manage animals with different behavioral responses within a herd are thus warranted. The more intuitive and perhaps utilized strategy to manage and ultimately shift the temperament of the cowherd towards a more adequate profile is genetic selection for docility and culling of aggressive animals. This strategy was more extensively utilized for *B. taurus* compared to *B. indicus*, due to many factors, including earlier domestication of taurine cattle and greater intensity of human interaction, which led taurine cattle to being notably more docile than *B. indicus* [10,61,62].

Considering the different environments that each of the bovine subspecies is adapted to, it is reasonable that selection for temperament types had different objectives. *Bos indicus* cattle are usually reared in tropical, extensive systems with low human contact and high predator prevalence, thus intense selection for docility is contradictory with survival skills needed in such conditions [15]. *Bos taurus* cattle are better adapted to temperate regions, such as North America and Europe. In these regions, general conditions such as the climate (i.e., need for feed provision during winter) and even socio-economic structure (i.e., small cattle operations throughout Europe since the Middle Ages) typically foster more intense cattle-human interaction [61]. These and other factors most likely collaborated for an early perception of docility as a desirable trait in *B. taurus* beef cattle, accelerating the selection for a more adequate temperament compared to *B. indicus* breeds.

Docility is a moderately heritable trait in cattle [63]; thus, selection for temperament is an effective and impactful form to modify the profile of the herd and improve overall efficiency, avoiding the detrimental effects of excitable temperament described herein. In addition to direct genetic selection through culling and breeding, potential epigenetic effects on offspring temperament have recently been described. More specifically, offspring from dams that experience stress during gestation had more excitable temperament when compared to calves born from cows that were not experimentally submitted to stress during pregnancy [21]. These differences in temperament were more pronounced in female offspring, with heifers born from cows that were stressed during pregnancy showing greater temperament score, exit velocity, pen score, and serum concentrations of cortisol compared with control and male cohorts [21]. These outcomes are highly relevant to beef cattle, as longevity of replacement females is an important economic trait [64]. Excitable females are not only associated with impaired reproduction as described herein but will also tend to produce calves with undesirable temperament.

It is indisputable the efficiency and magnitude of the impact genetic selection can impose upon a population, and examples are abundant among many animal species [65,66]. However, even when practiced diligently, changing a phenotypical trait takes time, effort, organization, and may yield undesirable consequences if not performed ethically such as a reduction of the genetic pool within a breed or even a species [67]. Hence, additional strategies are needed to alleviate the negative impacts of temperament of beef cattle herds. The idea of animals becoming accustomed to specific events, procedures or interventions performed by humans is defined by the concept of habituation or acclimation, in which responsiveness to a stimulus is decreased by its repeated presentation to the animals [68]. Previous research investigating the efficacy of habituating cattle to human handling has shown improvement in behavioral responses associated with temperament and alleviation of neuroendocrine stress response in animals exposed to frequent human interaction. Cows previously acclimated to physical restraint also had reduced plasma concentrations of cortisol and improved release of gonadotropin hormones [69].

Following that rationale, our group has hypothesized that acclimating beef females to handling procedures could potentially ameliorate the negatives effects of temperament and stress on reproduction. A series of studies was conducted with the objective of evaluating puberty attainment and reproductive success of cattle that were acclimated vs. non-acclimated to processing facilities and human interaction [30,33,34]. Acclimating crossbred mature *B. indicus* × *B. taurus* beef cows over two consecutive years yielded inconsistent, yet promising results (Table 3). Briefly, significant interactions between year of study, body reserve dynamics, reproductive performance, and breed types (Braford and ¼ Brahman × ¾ Angus cows were utilized) prevented more elucidating conclusions [34]. Nonetheless, acclimated Braford cows in the first year of study did show improved fertility, suggesting potential effects of acclimation in reproductive performance.

In another attempt to understand the effects of acclimation on reproductive outcomes, *B. indicus* × *B. taurus* heifers of similar genetic composition of cows from the previous study were utilized (Table 3), again over two consecutive years 33]. In this second study, more pronounced effects of acclimation on reproductive performance were reported. Acclimated heifers showed hastened attainment of puberty compared to non-acclimated cohorts despite reduced growth rate; a known key regulator of puberty attainment in beef heifers [57]. Based on these results and previous literature [70,71,72] it is speculated that younger cattle may respond better to habituation procedures in comparison to older cohorts. Another divergent aspect between these two studies [33,34] is the acclimation procedure utilized. While the mature cows were exposed only to human presence—i.e., a handler would walk among cows twice a week in the acclimated group, while the control group was left undisturbed [34]—the acclimated heifers were brought to handling facilities and processed, three times weekly [33]. Perhaps the more intense habituation protocol imposed to the heifers was more effective in habituating the animals and reducing their neuroendocrine response in comparison to the milder approach applied to mature cows. Similarly, *B. taurus* beef heifers also benefited from habituation [30]. Plasma cortisol concentrations and exit velocity were reduced and puberty attainment was hastened in acclimated heifers during the experimental period compared to control cohorts (Table 3). Collectively, these results corroborate that acclimating pre-pubertal beef females to handling procedures alleviates the neuroendocrine response, improves behavioral responses associated with temperament, and enhances the reproductive development of beef heifers across subspecies. Additionally, there is evidence suggestive of similar benefits in crossbred mature beef cows, although in older animals subjected to more subtle acclimation protocols these benefits are not as pronounced.

## 5. Conclusions

This review describes a series of research studies demonstrating the impacts of temperament on reproductive success and overall productivity of beef females. Research by our group was the first to document the importance of cattle temperament to overall production of cow-calf systems [30,31], and similar outcomes have since been reported by others [73,74]. One of the mechanisms causing these effects is the increased stimulation of the neuroendocrine response, mounting a stress reaction that culminates with increased release of cortisol into the bloodstream. This cascade affects cattle fertility through interactions with gonadotropin hormones, pregnancy establishment, and puberty attainment events. Genetic selection has the potential to impact cattle temperament due to the moderate heritability of this trait. Epigenetic effects of pre-natal stress have also been described to alter the temperament of the offspring. Acclimation to human handling, facilities, and routine are a management alternative to manage herd temperament, which seizes the benefits described for cattle with adequate temperament when compared to excitable ones. Hence, utilizing selection and management strategies to improve general herd temperament and behavior is beneficial for the overall efficiency and productivity of beef operations. Nonetheless, research should continue efforts to further understand the complex interactions between temperament, behavior, and physiology in beef cattle.

## Figures and Tables

**Table 1:** Concentrations of plasma, serum, and hair cortisol in beef cattle classified according to their temperament type. In all identified studies that followed the selection criteria ^1^ (n = 6) ^2^, cattle with more excitable temperaments ^3^ have significantly higher plasma or serum concentrations of cortisol. No differences between treatment types were identified for concentrations of cortisol in hair, a marker of chronic stress in beef cattle [23]. This may suggest that excitable cattle have similar basal concentrations of cortisol compared to cattle adequate temperament.

Item	Adequate	Excitable	*p*-Value	Type of Cattle
Serum cortisol, ng/mL [27]	26.1 (tame; n = 10) 32.3 (normal; n = 9)	53.7 (wild; n = 5)	<0.02	*B.indicus* heifers (Brahman)
Plasma cortisol, ng/mL [28]	30.5 (n = 12)	46.3 (n = 12)	<0.01	*B. taurus* steer calves (Angus × Hereford)
Serum cortisol, ng/mL [29]	10.18 (calm; n = 22) 11.91 (intermediate; n = 79)	14.99 (n = 28)	<0.01	*B. taurus* steers (Bonsmara × Romosinuano and Angus)
Plasma cortisol, ng/mL [30]	17.8 (n = 324)	22.7 (n = 109)	<0.01	*B. taurus cows* (Angus × Hereford)
Serum cortisol, ng/mL [31]	39.1 (n = 726)	49.1 (n = 227)	<0.01	*B.indicus* cows (Nelore)
Hair cortisol, pg/mg hair [31]	4.31 (n = 726)	4.23 (n = 227)	0.91	*B.indicus* cows (Nelore)
Plasma cortisol, ng/mL [32]	35.8 (n = 96)	50.8 (n = 74)	<0.01	*B.indicus* heifers (Nelore)

^1^ Studies must meet all criteria: breed of cattle must be considered primarily for beef production; must have classified animals according to their temperament using a described method; must have measured blood concertation of cortisol (plasma or serum) and must have reported results separately for temperament types. Many other studies have reported association between temperament and cortisol concentrations in beef cattle [33,34,35,36]. ^2^ Although a comprehensive search was performed in an effort to identify all eligible studies, these identified bodies of data may not represent the totality of published work that meets the specified criteria. ^3^ Definitions of excitable were consistent with the concepts of temperament score utilized herein and previously by our group [4] for temperament assessment, although they were not identical. Refer to cited studies for more details.

**Table 2:** Productive and reproductive variables evaluated in *B. indicus* and *B. taurus* cows in two separate studies. In both studies cows were classified according to their temperament type into adequate (temperament score ^1,2^ ≤ 3) or excitable (temperament score ^1,2^ > 3), utilizing chute score, and exit velocity.

Item	Adequate	Excitable	*p*-Value
*B. indicus* [31]	n = 726	n = 227	
Pregnancy rates, % ^3^	47.3	41.0	0.09
Pregnancy loss, % ^4^	5.9	9.9	0.05
Calving rate, %	74.8	68.3	0.04
Weaning rate, %	69.4	63.9	0.09
Calf weaning BW, kg	210	204	0.04
Kg of calf weaned per cow exposed, kg	146	130	0.04
*B. taurus* [30]	n = 324	n = 109	
Pregnancy rates, %	94.6	88.7	0.03
Pregnancy loss, % ^5^	2.83	3.74	0.63
Calving rate, %	91.8	85	0.04
Weaning rate, %	89.9	83.9	0.09
Calf weaning BW, kg	248	247	0.71
Kg of calf weaned per cow exposed, kg	223	207	0.08

^1^ Temperament score is calculated as an average of chute score and exit score. Chute score is evaluated in a 1 to 5 scale (1 = calm with no movement; 2 = restless movements, 3 = frequent movement with vocalization, 4 = constant movement, vocalization, shaking of the chute, and 5 = violent and continuous struggling) and exit score is evaluated by assigning a score from 1 to 5 according to animal placement within exit velocity quintiles (1 = cattle within the slowest quintile; 5 = cattle within the fastest quintile). ^2^ This criterion was developed based on research from our group, suggesting that moderate temperament (such as temperament scores 2 and 3) does not substantially impair production traits. ^3^ Pregnancy rates to the first insemination event. ^4^ Pregnancy loss was calculated based on pregnancy diagnosis 45 d after the breeding season and calving rates. ^5^ Pregnancy loss was calculated based on pregnancy diagnosis (120 to 180 days after the end of breeding season) and calving rates.

**Table 3:** Effects of acclimation to human handling on temperament and productive traits of beef cattle [4,30,33,34] ^1^.

Item	Acclimated	Non-Acclimated	*p*-Value
*B. indicus × B. taurus* cows [34]	(n = 197) ^2^	(n = 197) ^2^	
Plasma cortisol, after acclimation ng/mL	33.3	33.6	0.88
Chute score after acclimation	1.98	1.96	0.59
Body weight change, kg	−43	−50	0.38
*B. indicus × B. taurus* heifers [33]	(n = 40)	(n = 40)	
Plasma cortisol after acclimation, ng/mL	37.8	50.5	<0.01
Chute score after acclimation	1.37	1.84	<0.01
Growth rate until breeding season, kg/d	0.50	0.58	<0.01
*B. taurus* heifers [30]	(n = 44)	(n = 44)	
Plasma cortisol after acclimation, ng/mL	26.1	32.8	0.01
Growth rate until breeding season, kg/d	0.47	0.46	0.37
Exit velocity at breeding season, m/s	2.10	2.56	0.02

^1^ Cattle were assigned or not assigned to a 28-d human acclimation process within 45 d after weaning. Acclimated cattle were processed through a handling facility (heifers = 3× weekly, steers = twice weekly) for 4 weeks, whereas non-acclimated cohorts remained undisturbed on pasture. Cattle temperament was evaluated via chute score and exit velocity. Exit velocity was divided into quintiles and cattle assigned with a score from 1 to 5 (exit score: 1 = slowest animals; 5 = fastest animals). Individual chute and exit scores were averaged for calculation of temperament score. ^2^ Cows were divided in groups, which were experimental units. For more information, refer to Cooke et al. [31].
